# Calcium-dependent protein kinase CPK31 interacts with arsenic transporter AtNIP1;1 and regulates arsenite uptake in *Arabidopsis thaliana*

**DOI:** 10.1371/journal.pone.0173681

**Published:** 2017-03-15

**Authors:** Ruijie Ji, Liming Zhou, Jinglong Liu, Yuan Wang, Lei Yang, Qinsong Zheng, Chi Zhang, Bin Zhang, Haiman Ge, Yonghua Yang, Fugeng Zhao, Sheng Luan, Wenzhi Lan

**Affiliations:** 1 College of Resources and Environmental Science, Key Laboratory of Marine Biology, Nanjing Agricultural University, Nanjing, Jiangsu, China; 2 College of Life Sciences, Fujian Agriculture and Forestry University, Fujian, Fuzhou, China; 3 State Key Laboratory for Pharmaceutical Biotechnology, Nanjing University-Nanjing Forestry University Joint Institute for Plant Molecular Biology, College of Life Sciences, Nanjing University, Nanjing, China; 4 Department of Plant and Microbial Biology, University of California, Berkeley, CA, United States of America; Iwate Ika Daigaku, JAPAN

## Abstract

Although arsenite [As(III)] is non-essential and toxic for plants, it is effectively absorbed through various transporters into the roots. Here we identified a calcium-dependent protein kinase (CPK31) response for As(III) tolerance in *Arabidopsis*. We identified CPK31 as an interacting protein of a nodulin 26-like intrinsic protein (NIP1;1), an aquaporin involved in As(III) uptake. Similarly to the *nip1;1* mutants, the loss-of-function mutants of *CPK31* improved the tolerance against As(III) but not As(V), and accumulated less As(III) in roots than that of the wild-type plants. The promoter-*β*-glucuronidase and quantitative Real-Time PCR analysis revealed that *CPK31* displayed overlapping expression profiles with *NIP1;1* in the roots, suggesting that they might function together in roots. Indeed, the *cpk31 nip1;1* double mutants exhibited stronger As(III) tolerance than *cpk31* mutants, but similar to *nip1;1* mutants, supporting the idea that CPK31 might serve as an upstream regulator of NIP1;1. Furthermore, transient *CPK31* overexpression induced by dexamethasone caused the decrease in As(III) tolerance of transgenic *Arabidopsis* lines. These findings reveal that CPK31 is a key factor in As(III) response in plants.

## Introduction

Arsenic (As) is a metalloid and present as pentavalent arsenate [As(V)] and trivalent arsenite [As(III)] that reversibly convert depending on the redox status of the environment. Typically, As(V) and As(III) are the prevalent form in aerobic or anaerobic soils, respectively[1]. Although As is a non-essential element in plants, it can accumulate in crop plants, specially rice, and deposit into grains when the soil and water are contaminated [2,3]. Crops exhibit a range of As toxicity symptoms that are detrimental to growth and yield once As accumulation reaches beyond an optimal level, and may eventually pose a potential health risk to their consumers including animals and humans [3]. Due to its toxicity, universal distribution, and tendency to accumulate in animals and humans, As is ranked as one of the most toxic elements and defined as a group I carcinogen [4,5].

As(V), as a phosphate analogue, has a strong affinity with iron oxides or hydroxides in the soil, and its concentration is normally under 2.3 μM even in a highly As-contaminated soil [6,7]. As(V) thus enters plant roots mainly through the high-affinity phosphate transporters, such as PHT1;1 in *Arabidopsis* and OsPT8 in rice [8,9]. After acquisition, As(V) is rapidly reduced to As(III) by the enzyme arsenate reductase, and As(III) consequently acts as the predominant form of As for As extrusion, long-distance transport and sequestration in plants [4,10]. As(III) is uncharged at neutral pH in flooded paddy soils, and is more soluble and mobile compared with As(V). As(III) enters the cells mainly through nodulin 26-like intrinsic proteins (NIPs) subfamily of aquaporin proteins that belong to the major intrinsic proteins (MIPs), a family essentially facilitates the diffusion of water and small uncharged solutes in all domains of life [4,10–11]. Plant NIPs are remarkably well conserved across species, and can be divided into five well-defined subgroups (specified as NIP1-NIP5) according to phylogenetic analysis [11]. In addition to As(III), NIPs are also involved in the uptake and extrusion of other metalloids, including antimonite, boron, silicon and selenium, into or out of the plants and distribution within the plant body [11,12].

Several NIPs are permeable for As(III). Exogenous expression of AtNIP1;1 enhanced the As(III) uptake into the oocytes, as did its close homologue AtNIP1;2, and OsNIP1;1 and OsNIP3;1 from rice [2, 13]. In addition, NIP1;2, NIP3;1, NIP5;1, NIP6;1, and NIP7;1 of *Arabidopsis*, OsNIP2;1 and OsNIP3;2 from *Oryza sativa*, HvNIP1;2 from barley, and LjNIP5;1 and LjNIP6;1 from *Lotus japonicas* are all permeable to As(III) in the yeast expression system [14–16]. Among nine members of the NIP subfamily in *Arabidopsis*, NIP1;1 was firstly identified as a determinant of As(III) tolerance using a forward genetics screen [13]. NIP1;1 is highly expressed in roots and localized at the plasma membrane, and its loss-of-function mutants displayed an As(III)-tolerant phenotype with strong growth of roots [13], indicating that NIP1;1 is a critical As(III) transporter that mediates toxic As(III) uptake into roots. However, little is known about potential signaling mechanisms that allow roots to regulate NIP1;1 activity in response to As(III) toxicity.

GmNod26, a NIP family member abundantly targeted to the symbiosome membrane of nitrogen-fixing nodules of soybean roots, has been shown to be phosphorylated by a symbiosome membrane-associated calcium-dependent protein kinase (CPK) on Ser-262 of C-terminal [17, 18]. CPKs contain calmodulin-like Ca^2+^-binding domains and a kinase domain in a single protein, and have therefore been recognized as the major transducers of calcium signals and catalyze the phosphorylation of multiple proteins, including membrane channels, pumps, and a number of metabolic enzymes [19]. More studies have shown that several members of CPKs physically associate with ion channels and transporters to effectively regulate their activities [20–24]. Therefore, it is conceivable that CPKs fulfills critical functions in regulating the activity of NIP1;1 involved in As(III) uptake in the roots.

In the present study, we report the identification of CPK31 as a major component controlling As(III) tolerance in *Arabidopsis*. Our genetic and biochemical studies indicate that CPK31 fulfils this function by interaction with NIP1;1, providing a novel role of CPK31 in controlling As(III) toxicity in plants.

## Materials and methods

### Plant materials and growth conditions

*Arabidopsis thaliana* wild-type and the T-DNA insertion mutants *nip1;1–1* (SALK_016617), *nip1;1–2* (SALK_017916), *cpk31-1* (SALK_049228), *cpk31-2* (SALK_007777) and *cpk31-3(*SALK_049236) were obtained from the *Arabidopsis* Biological Resource Center. The ecotype of *Arabidopsis thaliana* lines is Columbia-0 (Col-0). Homozygous individuals were identified by RT-PCR using the primers listed in S1 Table. To obtain the *cpk31 nip1;1* double mutants, *cpk31-1* and *nip1;1–1* mutants were crossed. We identified *cpk31 nip1;1* double mutants by antibiotic selection and RT-PCR analysis using the primers listed in S1 Table.

Surface-sterilized seeds were plated on half-strength MS medium containing various sodium arsenite concentrations with 1% (w/v) sucrose and pH was adjusted to 5.7, and solidified using 0.8% (w/v) agar. Seedlings were grown on vertically placed plates in the chambers at 23–24°C with light intensity of 150 μmol·m^−2^·s^−1^ and 16:8 h light/dark photoperiods. The seedlings grown for 14 days after germination were imaged for measuring primary root lengths of plants using the ImageJ software (http://rsb.info.nih.gov/ij/), and then were collected for weighing the whole-plant biomass. For soil culture, 3-day-old seedlings germinated on half-strength MS medium were carefully removed to the nutrient-rich soil. For hydroponic culture, 7-day-old seedlings grown on half-strength MS medium were transferred into liquid 1/6 MS medium for further growth. The soil and hydroponic cultured plants were grown in the greenhouse under 150 μmol·m^−2^·s^−1^ light intensity with a 16-h light/8-h dark photoperiod at 22°C.

### Yeast-two-hybrid assay

Cytoplasmic C-terminal region of *NIP1;1* and the *CPK31* full-length cDNA were cloned into the activation domain vector pGADT7 and the DNA-binding domain vector pGBKT7. The recombinant vectors were transformed into yeast strain AH109 by the lithium acetate method reported as previous [24–27]. Transformants were selected the synthetic complete agar medium (SC) minus leucine and tryptophan and grown at 30°C for 4 d, and then transferred on SC minus histidine, leucine and tryptophan, or SC minus adenine, histidine, leucine and tryptophan supplemented with 10 mM 3-amino-1,2,4,-triazole and 40 μM X-*α*-gal. For serial dilution assay, exponentially grown yeast cells were harvested and adjusted to OD_600_ = 0.5 with sterilized double-distilled water and diluted to 1/10, 1/100, and 1/1000. Each yeast two-hybrid assay has been independently repeated at least three times. All primers used in this study are summarized in S1 Table.

### Subcellular location and bimolecular fluorescence complementation (BiFC) Assay

For subcellular location of CPK31 and NIP1;1, the coding sequence of *CPK31* and *NIP1;1* was respectively amplified without the stop codon from wild-type *Arabidopsis* cDNA and subcloned into pEZS-NL vector. To generate the BiFC constructs, *NIP1;1* and *CPK31* full length cDNA with no stop codon were subcloned into 35S-SPYNE(R)173 and 35S-SPYCE(M) vectors, respectively [28]. For transient expression, constructs were transformed into protoplasts of *Arabidopsis* suspension cultured cells according to the described methods [29]. For microscopic analyses, the transfected protoplasts incubated after 16 h at 22°C were imaged by confocal microscopy (LSM 710, Zeiss) equipped with an argon/krypton laser. The excitation wavelengths for GFP and YFP signals were 488 and 514 nm, respectively.

### Measurements of As (III) content

Three-week-old seedlings grown under liquid 1/6 MS medium were transferred for the solutions containing 10 μM As(III). After 0 h, 12 h, 24 h, 48 h and 6 days of incubation, the plants were washed three times with distilled water. The shoots and roots were collected and dried for 72 h at 80°C. After weighed, the samples were digested in ultrapure HNO_3_ (Sigma-Aldrich). As(III) contents were determined using the inductively coupled plasma Atomic Fluorescence Spectrometry (PerkinElmer). As(III) contents in the samples were calculated as the ratio of values to dry weights.

### Histochemical GUS analysis

For histochemical analysis of *CPK31*, a 2004-bp fragment upstream of starting codon was amplified with the primers listed in S1 Table, and subcloned into the pBI-101.1 binary vector. The *Agrobacterium tumefaciens* cells (GV3101) carrying the recombinant vector were used to transform *Arabidopsis* ecotype Col-0 plants with a floral dip method described previously [30]. Transformed *Arabidopsis* lines were selected based on kanamycin resistance. The GUS staining was done according to published protocols [31] with slight modification. In brief, T3 transgenic seedlings were incubated in GUS staining solution (2 mM 5-bromo-4-chloro-3-indolyl-*β*-D-glucuronide, 1 mM K_3_(Fe(CN)_6_), 1 mM K_4_Fe(CN)_6_·3H_2_O, 10 mM Na_2_EDTA, 0.1% Triton X-100, and 50 mM Na_3_PO_4_, pH 7.00) at 37°C for 12 h. After sufficiently decolorized with 75% (vol/vol) ethanol, individual representative plant tissues were photographed with an Olympus SZX12 microscope equipped with a camera.

### Quantitative real-time PCR analysis

Total RNA was extracted from the plantlets grown in the hydroponic system using the TRIzol reagent (Invitrogen) following the manufacturer’s instructions. The first-strand cDNA was synthesized by M-MLV Reverse Transcriptase (Promega) with anchored oligo(dT_18_). Quantitative real-time PCR was performed on a CFX Connect Real-ime System (Bio-Rad) using the QuantiFast SYBR Green PCR Kit (Qiagen). All primers for expression assays were listed in S1 Table. The expression value of every sample was quantified with the 2^−ΔΔCT^ method [32] using *ACTIN2* as an internal reference.

### Construction of dexamethasone-inducible transgenic *Arabidopsis* lines

For the inducible expression of *CPK31*, the full-length *CPK31* CDS was amplified by PCR from a wild-type cDNA pool using specific primers and then was cloned into the binary vector pTA7002. The generated construct was further sequenced for confirmation and introduced into *Agrobacterium tumefaciens* (strain GV3101) cells for transformation into the *cpk31-1* mutant plants. More than 50 independent lines were selected for hygromycin resistance. Homozygous transformants were confirmed by both segregation and PCR analysis. Three representative lines were used for phenotypic analysis and molecular characterization. The seeds were planted at half-strength MS medium containing 10 μM dexamethasone (DEX). 7-day-old seedlings were gathered for photograph and analysis of *CPK31* expression and root length.

## Results

### CPK31 physically interacts with NIP1;1

**Fig 1:**
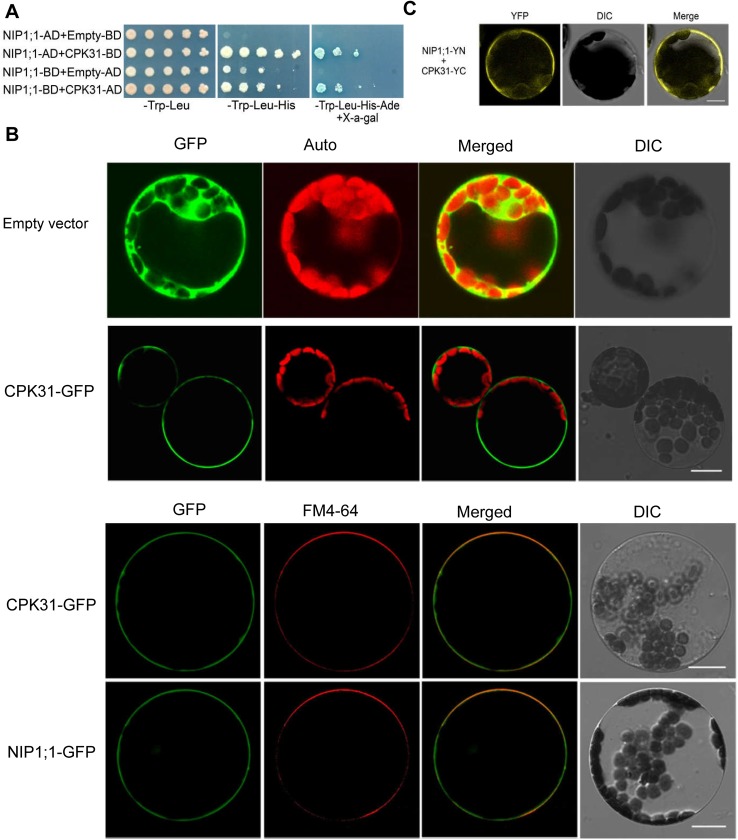
Interactions between CPK31 and NIP1;1 at the plasma membrane. **(*A*)** Yeast two-hybrid assay of the interactions between CPK31 and NIP1;1. Yeast cells were cotransformed with various combinations of pGBT9- and pGADGH-fusion constructs as indicated on the left of each row. Serial decimal dilutions of corresponding yeast cells were spotted on the synthetic complete agar medium (SC) minus leucine and tryptophan (-Trp-Leu; left lane), SC minus leucine, tryptophan and histidine (-Trp-Leu-His; middle lane), or SC minus leucine, tryptophan, histidine, adenine and contained 40 μM X-*α*-gal (-Trp-Leu-His-Ade; right lane). Photographs were taken after cultivation at 30°C for 4 days. (***B***) Subcellular location of CPK31-GFP and NIP1;1-GFP in *Arabidopsis* cells. The GFP coding sequence was fused to the *C*-terminus of CPK31 or NIP1;1 coding sequence without stop codon in the pEZS-NL vector, and then transformed into protoplasts of *Arabidopsis* suspension cultured cells. Empty vector was transformed as a control. To further conform potential plasma membrane location, the protoplasts were stained with the plasma membrane-specific dye FM4-64 (red). **(*C*)** CPK31::35S-SPYCE co-expressed with NIP1;1::35S-SPYNE in plasma membrane of *Arabidopsis* protoplasts observed by bimolecular fluorescence complementation (BiFC). CPK31::35S-SPYCE (CPK31-YC) and NIP1;1::35S-SPYNE (NIP1;1-YN) were transformed into the protoplasts isolated from *Arabidopsis* suspension cultured mesophylls. The fluorescence signals shown at (***B***) and (***C***) were imaged under a Zeiss confocal microscope as green and yellow, respectively (Scale bar: 10 μm).

CPKs act as major calcium sensors and transducer, and play a fundamental role in growth, development and stress responses in plants. We have established a yeast two-hybrid system and successfully identified a number of interaction partners between the kinases and ion channels [24–27]. We hypothesized that some of CPKs may interact with and regulate the activity of AtNIP1;1 involving in As(III) uptake in the roots. To test this possibility, we thus screened all CPK members in *Arabidopsis* to find potential candidates physically interacting with AtNIP1;1 by using the yeast two-hybrid assay. Among the 34 members of CPKs tested, we found that only CPK31 (AT4G04695) interacted physically with the putative cytoplasmic hydrophilic domain at N-terminal of AtNIP1;1 (Fig 1A).

CPK family members were detected at multiple subcellular locations, including cytosol and plasma membrane [19]. To determine the subcellular localization of CPK31 in the *Arabidopsis* cells, we transiently expressed a CPK31-GFP fusion protein in *Arabidopsis* protoplasts. The CPK31-GFP signal appeared at the plasma membrane, while the control GFP was localized to the cytoplasm under confocal laser scanning microscopic analysis (Fig 1B). Moreover, CPK31-GFP green signal was colocalized with the red signal of FM4-64, a plasma membrane-specific dye (Fig 1B), confirming that CPK31 appears in the plasma membrane. This result is consistent with the prediction of membrane subcellular location of CPK31 with a myristoylation motif at N-terminal [19]. Similarly, GFP-tagged AtNIP1;1 signal appeared at the plasma membrane and overlapped with the FM4-64 signal (Fig 1B), exhibiting plasma membrane localization at the mesophyll protoplast, consistent with the observation in the root epidermal cells of the transgenic *Arabidopsis* lines [13].

To further confirm that the interaction between CPK31 and AtNIP1;1 could occur in plant cells, bimolecular fluorescence complementation (BiFC) assay was performed in protoplasts of suspension cultured *Arabidopsis* mesophyll. The fluorescence signals were primarily detected at plasma membrane of the cells co-expressing AtNIP1;1 and CPK31, while no fluorescence signals were detected in the cells expressing the fusion of AtNIP1;1-SPYNE or CPK31-SPYCE (Fig 1C). Therefore, these results suggest that the interaction between of CPK31 and AtNIP1;1 primarily occurs at the plasma membrane of plant cells.

### The *cpk31* mutants are more tolerant to As (III) than the wild-type plants

Work by Kamiya et al (2009) demonstrated that NIP1;1 plays a negative role in plant survival under high-As(III) conditions, because the *nip1;1* loss-of-function mutants are more As-resistant than the wild-type [13]. It is believed that NIP1;1 mediates As(III) uptake into the roots thus contributes to the toxicity of external As(III). However, the mechanisms for activating NIP1;1 activity are still unknown. Some CPKs have been shown to activate channels by interacting with the channel proteins [20,21,23,24], and some inhibit the activity of channels [22]. Based on our findings of interaction of CPK31 and AtNIP1;1, we tested the possible regulatory mechanism of CPK31 on AtNIP1;1 by using transgenic *Arabidopsis* lines.

**Fig 2:**
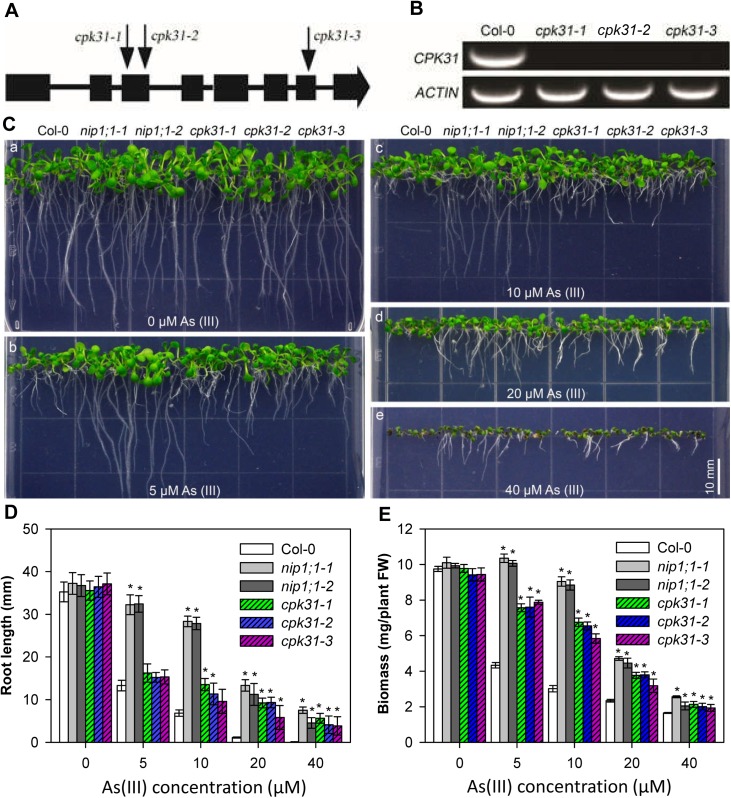
Isolation of *cpk31* T-DNA insertional mutants and As (III) resistance assays. **(*A*)** Scheme of the *Arabidopsis CPK31* gene structure and schematic T-DNA insertion sites of *Arabidopsis* lines SALK_049228, SALK_007777 and SALK_049236. Solid boxes and lines indicate exons and introns, respectively. The positions of T-DNA insertion in SALK_049228(*cpk31-1*), SALK_007777(*cpk31-2*) and SALK_049236 (*cpk31-3*) are indicated by arrows. **(*B*)** RT-PCR analysis of *CPK31* and *ACTIN2* mRNA levels in wild type (Col-0) and the three mutant lines (*cpk31-1*, *-2*, and -*3*). Three-week-old seedlings grown at half-strength MS were gathered for mRNA concentration analysis. **(*C*)** Comparative As(III) sensitivity analysis of wild-type, *nip1;1* and *cpk31* mutants. The seeds of wild-type (Col-0), *nip1;1–1*, *nip1;1–2*, and three *cpk31* mutant lines were plated at half-strength MS agar plates containing 0 (a), 5 (b), 10 (c), 20 (d), or 40 μM (e) As(III), and 8-day-old seedlings after germination were used for the photographs. Seedlings of wild-type (Col-0), *nip1;1–1*, *nip1;1–2*, and three *cpk31* mutant lines were plated on vertical half-strength strength MS agar plates containing 0 (a), 5(b), 10 (c), 20 (d), or 40 μM (e) As(III) for 8 days before taking the photographs. Scale bars = 10 mm. Length of primary roots **(*D*)** and whole-plant biomass **(*E*)** of wild-type (Col-0), two *nip1;1* and three *cpk31* mutant lines under various As(III) concentrations. The root length and biomass of 8-day-old seedlings cultured at the conditions described as **(*C*)** were measured (*n* >5 for each condition). Data are mean ± SD of four replicate experiments. Data are mean ± SD of four replicate experiments. Asterisks (*P< 0.05, Student’s *t* test,) represent statistically significant differences compared with Col-0 as the control.

We isolated and characterized T-DNA insertional alleles in the *CPK31* gene, including *cpk31-1* (SALK_049228), *cpk31-2* (SALK_007777) and *cpk31-3*(SALK_049236) (Fig 2A). *CPK31* mRNA was not detectable by RT-PCR in homozygous plants of either T-DNA allele (Fig 2B), indicating that T-DNA insertions inside the gene disrupted the expression of *CPK31* transcripts. To investigate the role of the *CPK31* gene in the regulation of As(III) tolerance, we used two As(III) toxicity resistant lines, *nip1;1–1* and *nip1;1–2* T-DNA inserted mutants used in the previous study [13], as positive controls. When grown on the normal medium without As(III), all *cpk31* and *nip1;1* mutant seedlings showed similar phenotype to the wild-type (Fig 2C). However, in the presence of 5 and 10 μM As(III), the growth of *cpk31-1*, *cpk31-2* and *cpk31-3* seedlings, although not as good as *nip1;1–1* and *nip1;1–2* mutant seedlings, was better than the wild-type, indicating CPK31 may regulate As(III) uptake under these conditions. With the increase in As(III) concentrations, the *cpk31* mutant seedlings displayed more similar phenotype to *nip1;1–1* and *nip1;1–2* mutant plants (Fig 2C–2E). Fig 2D and 2E respectively showed that root growth and biomass in the *cpk31-1*, *cpk31-2* and *cpk31-3* seedlings were significantly more tolerant to 20 and 40 μM As(III) treatment compared to the wild-type, and are similar to the *nip1;1* mutant plants. These findings support a model that CPK31 might interact with and regulate AtNIP1;1 activity in As(III) transport.

**Fig 3:**
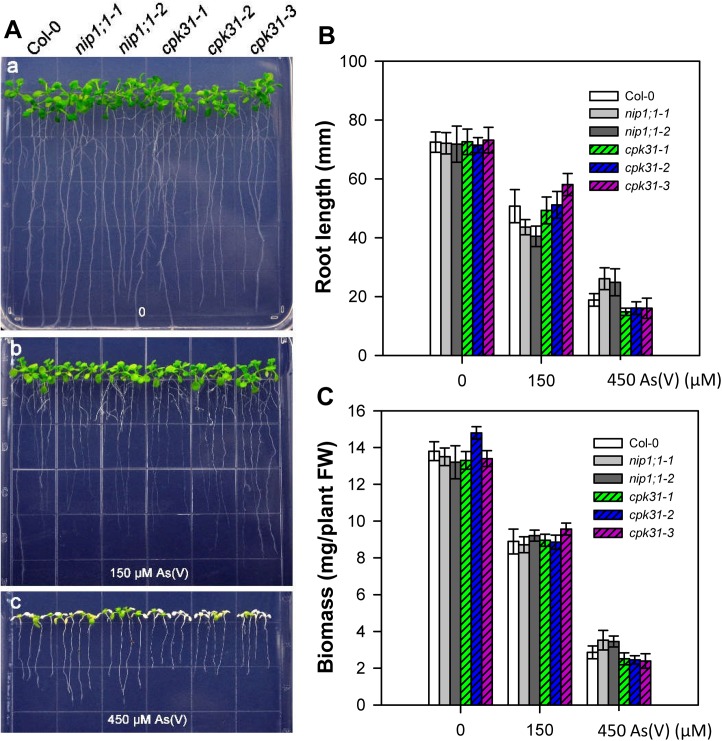
Growth phenotype of *cpk31* and *nip1;1* mutants under variable As(V) conditions. (***A***) Growth phenotype of 7-day-old wild-type (Col-0), *nip1;1–1*, *nip1;1–2*, *cpk31-1*, *cpk31-2*, and *cpk31-3* mutants in half-strength MS agar medium containing 0 (**a**), 150 (**b**), or 450 μM As (V) (**c**). Length of primary roots (**B**) and whole-plant biomass (**C**) of wild-type (Col-0), two *nip1;1* and three *cpk31* mutant lines grown for 10 days under 0, 150, or 450 μM As (V). Plants were grown at the normal half-strength MS for 3 days before transferred to the medium containing As(V). Data are mean ± SD of four replicate experiments. It was not statistically significant difference compared with Col-0 as the control (*P*< 0.05, Student’s t test).

Earlier studies have shown that As(V) and As(III) enter plant roots via two different systems: As(V) is taken up via phosphate transport family (PHT), while As(III) is transported by NIPs [6–8]. Furthermore, after absorbed into roots, As(V) was widely accumulated in the endodermis, the apex (meristem) and cap of roots, whereas As(III) was mostly accumulated in the endodermis as detected by X-ray fluorescence microscopy [33]. Considering that As(V) is one of the most present forms of As absorbed by roots from soils [7,8], we also tested whether CPK31 also participates in As(V) response in *Arabidopsis*. We analyzed the growth of *cpk31-3* mutants grown on half-strength MS medium containing various As(V) concentrations. As shown in Fig 3, *cpk31-1*, *cpk31-2* and *cpk31-3* seedlings showed similar growth to the wild-type under 0, 150, or 450 μM As(V), indicating that CPK31 may not regulate As(V) tolerance. Similarly, disruption of *NIP1;1* did not enhance survival against As(V) stress (Fig 3), supporting further the notion that CPK31 may function with NIP1;1 specifically in As(III) response.

### The *cpk31* mutants contain less As(III)

**Fig 4:**
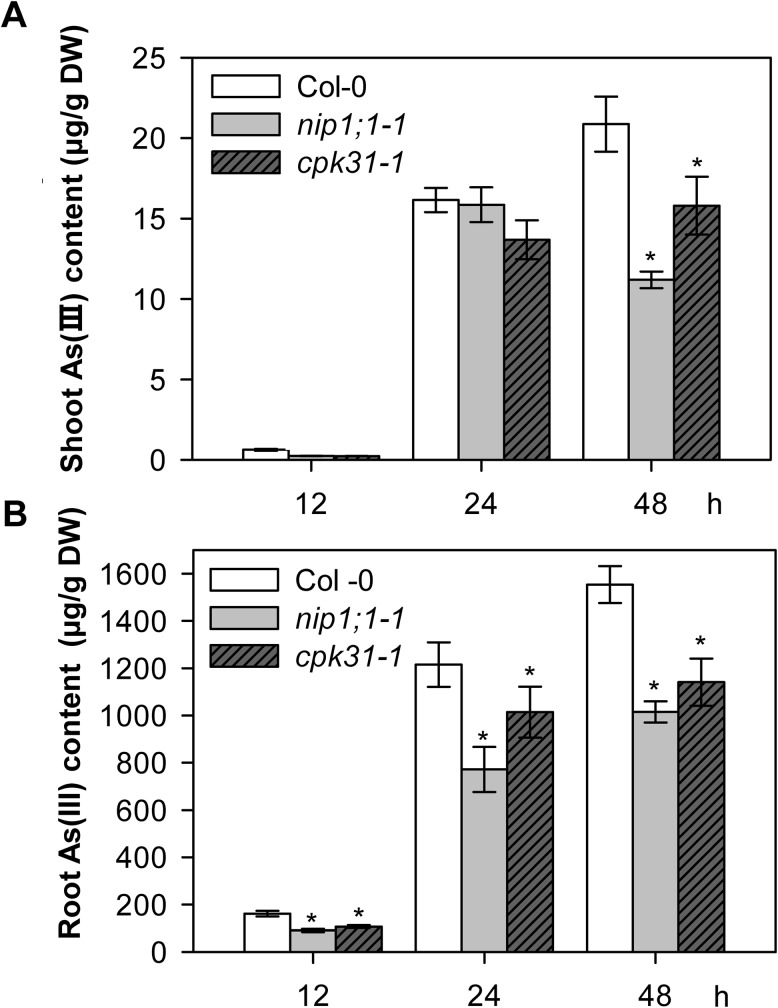
*cpk31-1* and *nip1;1–1* mutants contain less As (III) compared with the wild-type. As (III) contents in shoots (**A**) and roots (**B**) of seedlings under As(III) conditions. Three-week-old seedlings of wild-type (Col-0), *nip1;1–1* and *cpk31-1* mutant grown under the hydroponic medium were transferred to the medium containing 10 μM As (III) for 12, 24, or 48 h. Samples were collected at indicated time points after treatment and As(III) contents were determined. Summarized As(III) content in (A) and in (B) was respectively deduced from results of 5 seedlings/condition of two or four replicate experiments among three independent experiments. Data are mean ± SD, and asterisks indicate statistically significant difference between Col-0 and *nip1;1* or *cpk31* mutant plants (*P< 0.05, Student’s t test).

As(III) tolerance is determined by multiple processes, including acquisition and distribution. It was reported that As(III) tolerance in the *nip1;1* mutant lines resulted from the decrease in As(III) uptake from the environments [13,16]. If As(III) acquisition is altered in *cpk31* mutant plants through the AtNIP1;1 pathway, As(III) content in these plants may be reduced as well. Indeed, lower As(III) contents were observed in *cpk31-1* mutant roots as compared with the wild-type after exposure to As(III), although As(III) content in the *cpk31* mutants was still higher than the *nip1;1–1* mutants at 12, 24 and 48 h after As(III) exposure (Fig 4B). This result suggests that As(III) uptake and accumulation from the medium are less efficient in *cpk31* mutant plants.

We further determined whether CPK31 is involved in As(III) translocation between roots and shoots after As(III) exposure. Three-week-old *Arabidopsis* seedlings grown in the hydroponic medium were transferred to the medium containing 10 μM As(III). After 12 h and 24 h As(III) exposure, As(III) contents in shoots were not significantly different among wild-type, *nip1;1*, and *cpk31* mutants (Fig 4A), while those in the roots of *nip1;1*, and *cpk31* mutants were less than wild-type (Fig 4B). However, shoots of *nip1;1* and *cpk31* mutants accumulated much less As(III) than the wild-type when these seedlings were exposed to As(III) for 48h (Fig 4B). These results suggest that CPK31 distributes As(III) within *Arabidopsis* body, probably companies with NIP1;1, to decrease As(III) toxicity.

### CPK31 expression pattern overlapped with that of NIP1;1 in the roots

**Fig 5:**
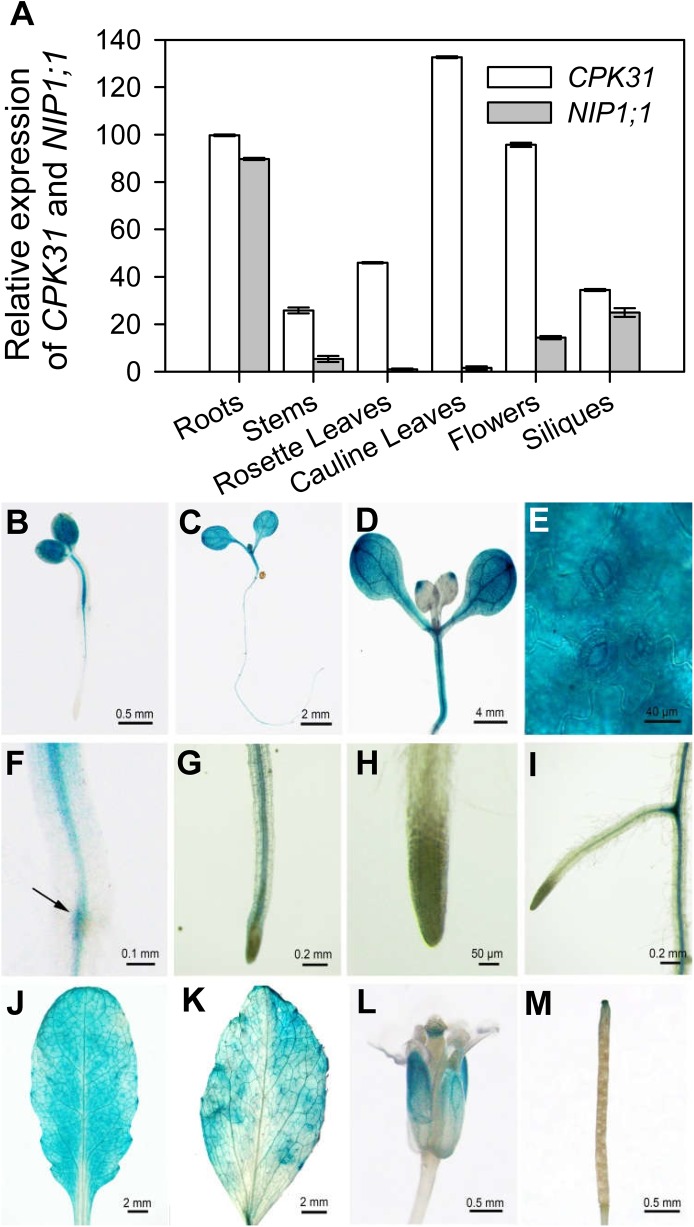
Analysis on tissue expression pattern of *CPK31*. (**A**) Quantification analysis of *CPK31* and *NIP1;1* transcripts in various organs of the wild-type plants. Total RNA was isolated from various tissues (root, leaf, stem, flower and silique) of 4-week-old wild-type plants (Col-0) grown in soil under long-day conditions. Values were normalized to *ACTIN2*, and the relative mRNA expression levels were calculated as the ratio of NIP1;1 or CPK31 mRNA level to the *NIP1;1* level in rosette leaves of plants (as 1.0). Data are mean ± SD of four replicate experiments. **(B-M)** Histochemical analysis of *CPK31* promoter–GUS expression in transgenic plants. GUS staining in 2-day-old (**B**), 3-day-old (**C**), and 7-day-old (**D**) transgenic seedlings grown on half-strength MS agar plates. The enlarged part of the cotyledon (**E**), root–hypocotyl junction (**F**), primary root (**G**), the tip of primary root (**H**), and lateral-primary root junction (**I**) of 7-day-old seedlings. GUS staining in cauline leaf (J), rosette leaf (**K**), flower (**L**), and silique (**M**) of mature *CPK31*::*GUS* transgenic plants. To obtain adult plants for staining, 7-day-old seedlings grown on 1/2 MS agar plates were transferred into soil, and samples used for GUS analysis were collected for (**J**) to (**M**) on the 21^th^ day after the transfer.

The results on protein-protein interaction and As(III) sensitivity suggest that CPK31 and NIP1;1 may function together in the same pathways. To gain further insights into the function of these proteins, we examined their expression pattern in plant tissues using qRT-PCR analysis. As shown in Fig 5A, the qRT-PCR analysis of gene transcripts indicated that *CPK31* was ubiquitously expressed in roots, stems, rosette leaves, cauline leaves, flowers and siliques of 4-week-old *Arabidopsis* plants. On the other hand, *NIP1;1* was expressed mostly in roots with low levels in other tissues, consistent with the earlier result that *NIP1;1* is preferably expressed in roots [13]. To further analyze the expression pattern of *CPK31*, we analyzed transgenic *Arabidopsis* plants expressing *CPK31* promoter-driving *β*-glucuronidase (GUS) reporter, *CPK31* promoter was active in cotyledons, rosette leaves, the vascular bundle of primary roots, and the junction of lateral roots and primary roots of 2-, 3-, and 7-day-old seedlings (Fig 5B–5I). In addition to expressed in primary roots, *CPK31* promoter the junction of primary root and lateral roots showed strong GUS activity (Fig 5I), It was noted that the activity of the *CPK31* promoter was very high in guard cells (Fig 5E), overlapping with the expression pattern of the *NIP1;1* genes [13]. We subsequently observed *CPK31* gene promoter in leaves of the four-week-old seedlings, and found that the activity of promoter was highly expressed in the vascular tissues of mature leaves with higher levels at cauline leaves (Fig 5J) than rosette leaves (Fig 5K), consistent with the qRT-PCR analysis (Fig 5A). Furthermore, *CPK31* was primarily expressed at petals and stigma of flower (Fig 5L), and the tip of siliques (Fig 5M).

**Fig 6:**
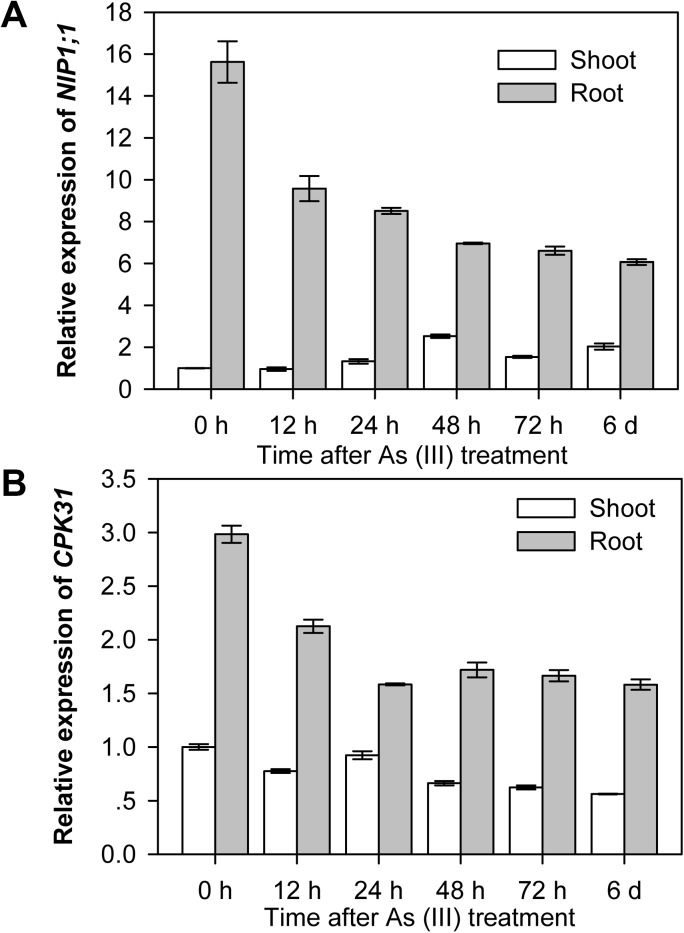
Expression of *CPK31* and *NIP1;1* is sensitive to As(III). The levels of expression of *NIP1;1*(***A***) and *CPK31*(***B***) in leaves and roots of the seedlings. 3-week-old plants grown at normal hydroponic medium were transferred to the medium with 10 μM As (III), and the roots and shoots of plants incubated for 0, 12, 24, 48, 72, or 144 h were gathered separately for mRNA measurement. After normalized to *ACTIN2*, the relative expression of *NIP1;1* or *CPK31* was calculated as the ratio of the normalized expression of *NIP1;1* or *CPK31* in shoot or root of seedlings treated with As(III) to that in shoot before treated with As(III). Data are mean ± SD of four replicate experiments with n = 3 for each experiment.

The assays described above indicated overlapping expression pattern of *CPK31* and *NIP1;1* in roots. We subsequently investigated whether mRNA levels of those two genes are regulated by As(III) treatment. After grown in normal hydroponic medium for 3 weeks, plants were transferred to the medium with 10 μM As(III). The roots and shoots of plants were collected at different time points and their mRNA levels were examined by qRT-PCR analysis. When exposed to As (III), both *CPK31* and *NIP1;1* mRNA levels in roots decreased within 12 hours (Fig 6). Interestingly, *CPK31* and *NIP1;1* in shoots responded to As(III) differently: *CPK31* mRNA levels stayed relatively stable, but the mRNA of *NIP1;1* increased. However, the mRNA levels of *CPK31* and *NIP1;1* altered more in roots than in shoots in response to As(III) (Fig 6). These results suggest that CPK31 and NIP1;1 might be functionally relevant, at least in roots, to decrease As(III) uptake under conditions of As(III) toxicity.

### As(III) sensitivity of *cpk31 nip1;1* double mutants and transient CPK31 overexpression lines

**Fig 7:**
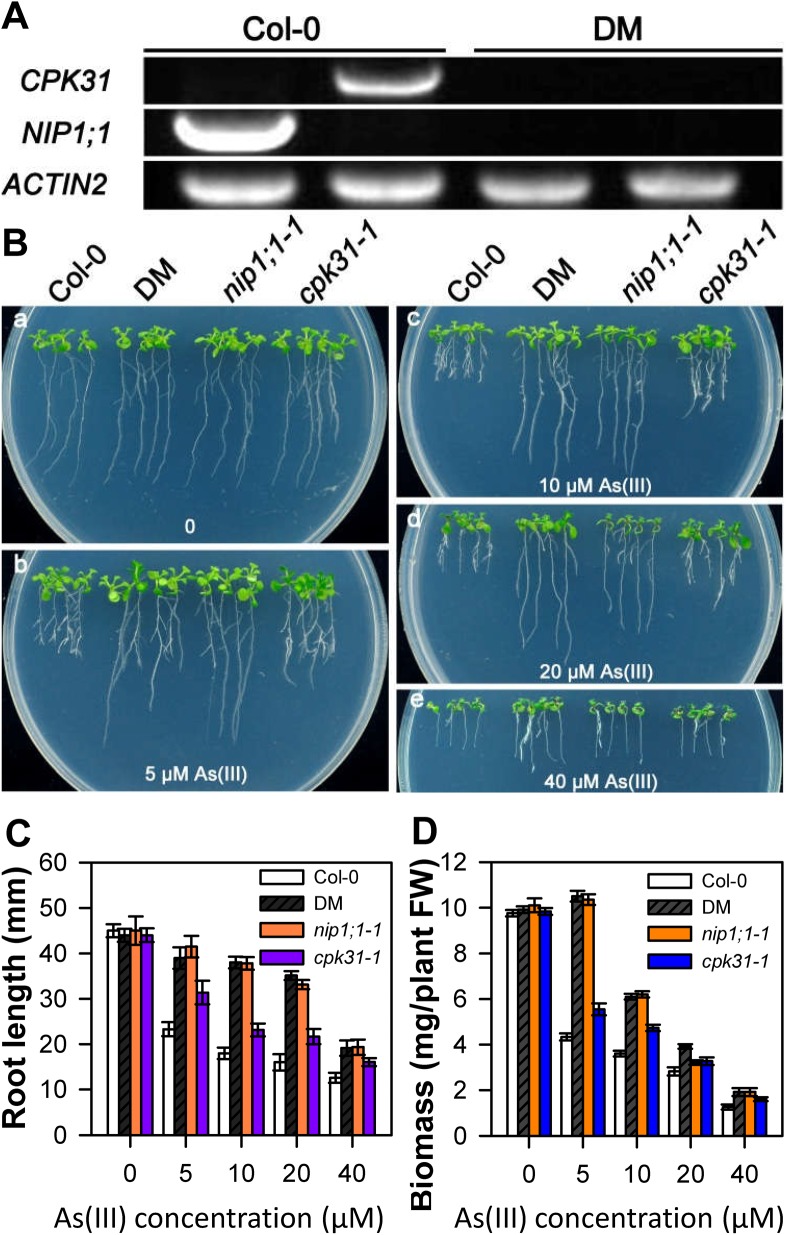
Growth phenotype of *cpk31 nip1;1* double mutants under variable As(III) conditions. (**A**) qRT-PCR analysis of *CPK31*, *NIP1;1* and *ACTIN2* gene expression from the wild-type (Col-0) and *cpk31 nip1;1* double mutants (DM). Expression of *ACTIN2* was analyzed as a quantitative control. (B) Growth phenotype of 10-day-old wild-type (Col-0), *cpk31 nip1;1* double mutants (DM), *nip1;1–1*, and *cpk31-1* mutants grown in half-strength MS agar medium containing 0 (**a**), 5 (**b**), 10 (**c**), 20 (**d**), or 40μM As(III) (**e**). The seeds of wild-type (Col-0), *cpk31 nip1;1* double mutants (DM), *nip1;1–1*, and *cpk31-1* mutants were firstly planted at the normal half-strength MS agar medium without As(III) for 4 days, and were then transferred for the medium containing various As(III) contents. Length of primary roots (**C**) and whole-plant biomass (**D**) of wild-type (Col-0), *cpk31 nip1;1* double mutants (DM), *nip1;1–1*, and *cpk31-1* mutants seedlings grown for 10 days under 0, 5, 10, 20, or 40 μM As(III). Data are mean ± SD of four replicate experiments. Asterisks indicate statistically significant difference between Col-0 and mutant plants exposed various contents of As (III) (*P< 0.05, Student’s t test).

CPK31 interacts physically with NIP1;1 and their genes are both expressed in roots. Together with the finding that both *nip1;1* and *cpk31* mutants showed similar phenotype in As(III) tolerance, we speculated that CPK31 and NIP1;1 might function in the same pathway. To address this hypothesis, we generated a *cpk31 nip1;1* double mutant from *cpk31-1* and *nip1;1–1* single mutants (Fig 7A). The homozygous plants of the *cpk31 nip1;1* double mutant did not produce detectable levels of transcripts of *CPK31* and *NIP1;1* as analyzed by RT-PCR, indicating disruption of both *CPK31* and *NIP1;1* expression.

To analyze whether CPK31 is one of crucial regulators for NIP1;1 activity, we examined the As(III)-tolerant phenotype of wild-type, *nip1;1*, *cpk31 nip1;1* double mutant seedlings grown on 1/2 strength MS agar plates under As(III) conditions. As shown in Fig 7, *cpk31 nip1;1* double mutants significantly enhanced root growth as compared with *cpk31* mutants, with longer primary root length and more biomass. Additionally, the growth phenotype of *cpk31 nip1;1* double mutants is similar to that of *nip1;1* mutants treated with As(III). Together, these results suggest that CPK31 might be one of factors for mediating NIP1;1 activity response for As(III) stress *in vivo*.

**Fig 8:**
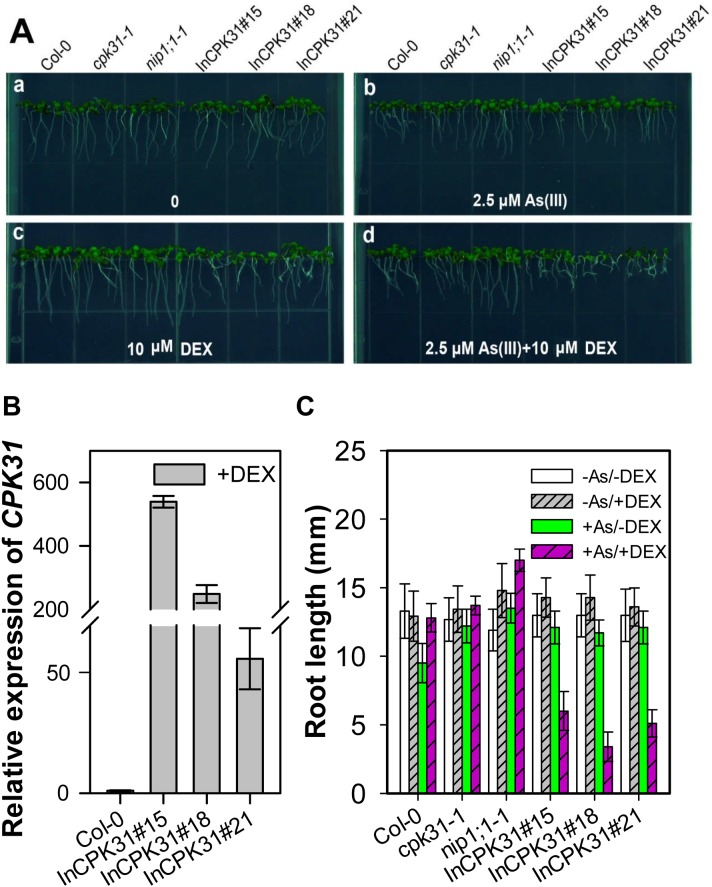
Dexamethasone (DEX)-induced transient *CPK31* expression caused a decrease in As(III) tolerance. (***A***) Growth phenotype of 7-day-old wild-type (Col-0), *cpk31-1*, *nip1;1–1*, *cpk31-1* mutant plants transformed with *CPK31* expression system (InCPK31) lines 15# (InCPK31#15), 18# (InCPK31#18), and 21# (InCPK31#21) in half-strength MS agar medium containing 0 (**a**), 2.5 μM As (III) (**b**), 10 μM DEX (**c**), or 2.5 μM As (III) and 10 μM DEX (**d**). (***B***) Relative expression level of *CPK31* in wild-type (Col-0), InCPK31#15, InCPK31#18, and InCPK31#21 lines treated with 10 μM DEX. The seedlings were obtained after 7 day DEX treatment for mRNA measurement. The relative expression of *CPK31* was calculated as the ratio of *CPK31* in InCPK31 lines to that in the wild type. The data are mean ± SD of three biological replicates. (***C***) The root length of the wild-type (Col-0), *cpk31-1*, *nip1;1–1*, InCPK31#15, InCPK31#18, and InCPK31#21 after 7 days of 10 μM DEX treatment. The data are mean ± SD of three biological replicates.

Because the knockout of *CPK31* caused an increase in As(III) tolerance, we tried to find whether overexpression of *CPK31* had a contrary response to As(III) in Arabidopsis. Thus, we constructed a dexamethasone (DEX)-inducible *CPK31* expression system (InCPK31) in the *cpk31-1* mutant background (Fig 8). We selected three lines of plants transformed with InCPK31, InCPK31#15, InCPK31#18, and InCPK31#21, as their *CPK31* expression was significantly induced by DEX (Fig 8B). The inducible expression system did not cause the changes in growth at the absence of DEX (Fig 8A and 8C). However, the root length of plants transformed with *CPK31* expression system (InCPK31), InCPK31#15, InCPK31#18, and InCPK31#21 was much shorter when these seedlings were grown at the medium containing DEX (Fig 8A and 8C), at which *CPK31* expression was significantly induced (Fig 8B). These results again supports the hypothesis that the negative roles of CPK31 in As(III) tolerance in *Arabidopsis*.

## Discussion

Calcium-dependent protein kinases (CPKs) are crucial signaling molecules that mediate responses to diverse endogenous and environmental cues. In the present study, we provide evidence that CPK31 appears to regulate As (III) uptake in roots through interaction with As (III)-permeable aquaporin NIP1;1. This finding provides evidences of key roles of CPK31 in the regulation of As(III) toxicity in plants.

CPKs comprise a large family of serine/threonine kinases in plants (34 genes in *Arabidopsis*) with the conserved Ca^2+^-binding EF hands in the calmodulin-like domain. This unique molecular structure allows that CPKs could be directly activated by Ca^2+^, and function as a Ca^2+^ sensor [19]. Some studies proposed that CPKs could be involved in As(V) toxicity to some extent [34]. However, whether CPKs are involved in As(III)-mediated signaling pathway and their potential functions in defending against As(III) toxicity remain to be discovered. We found that CPK31 was down-regulated by As(III) treatment (Fig 6), whereas several *CPKs* were up-regulated by As(V) stress in the rice roots by the transcriptomic analysis [34], suggesting that CPKs may have contrary roles in As(III) and As(V) tolerance. Three homozygous T-DNA-insert knockout lines of *CPK31* (*CPK31-1*, *-2* and *-3*) all had the enhanced As(III)-tolerant phenotype (Fig 2), and *cpk31-1* mutants accumulated As at a significantly lower rate in both roots and shoots than the wild-type plants (Fig 4). Furthermore, transient CPK31 overexpression induced by DEX caused the decrease in As(III) tolerance of transgenic *Arabidopsis* lines (Fig 8). These results indicate that CPK31 involving in As(III) uptake in roots and distribution in shoots.

As distinct Ca^2+^ sensor in plants, several CPKs, including CPK2, CPK13, CPK20, CPK21, CPK23, and CPK32, physically interacted with various channels [20–24]. Our results in this paper showed that CPK31 specifically interacts with NIP1;1 using yeast two hybrid screening, and used BiFC-based assays to confirm that the this interaction exists in *Arabidopsis* cells (Fig 1). CPK31 contains a myristoylation site at its N-terminus [19], known for targeting proteins to cellular membranes. The site could recruit CPK31 to the plasma membrane, where its anchoring target NIP1;1 lies (Fig 1)[13]. Therefore, it will be interesting to further disclose whether the existence of CPK31-involving Ca^2+^ signaling pathway for NIP1;1 functions.

CPK31 is mainly expressed at the vascular bundle but not root tips of the primary roots detected by histochemical staining of promoter-*CPK31*:*GUS* (Fig 5), with the similar patterns to NIP1;1 [13]. Moreover, RT-PCR analysis of *CPK31* and *NIP1;1* transcripts in root tissues indicated both expression of *CPK31* and *NIP1;1* was down-regulated once the roots were exposed to 10 μM As(III) (Fig 6). The decrease of *NIP1;1* and *CPK31* expression in presence of As(III) might result from the idea that NIP1;1 may not be "originally" designed to be As(III) carrier. For example, NIP1;1 also transports Sb(III) and determines the Sb(III) sensitivity of *A*. *thaliana* [12]. Thus, plants adapt to As(III) toxicity by developing this "avoidance" mechanism.

The overlapping expression profiles of *CPK31* and NIP1;1 in the roots are consistent with the interaction with each other. Interestingly, most As(III) was accumulated in these sites of roots exposed to As(III) [33]. The loss-of-function mutants of CPK31 improved the specific tolerance of roots against As(III) but not As(V) (Figs 2 and 3), and accumulated less As(III) in roots than those of the wild-type plants (Fig 4). It is noteworthy that *cpk31* mutants contained more As(III) with lower As(III) tolerance in the roots compared with *nip1;1* mutants, indicating that CPK31 might be one of the kinases involving in As(III) uptake by NIP1;1. This hypothesis is further supported by the observations that roots of *cpk31 nip1;1* double mutants showed stronger As(III) tolerance than *cpk31* mutants, whereas similar to *nip1;1* mutants (Fig 7). Moreover, as CPK31 is also present in other tissues such as the vascular tissues of leaves, stems and flowers, we speculate that it may function together with other components in those tissues as well, for example in the long-distance transport and distribution of As(III) throughout the whole plant, the functions of NIP1;1 was not involved [16].

As (III) is high toxic to organisms, due to its high affinity to bind with sulfhydryl groups of many proteins and then disrupting many key metabolic processes in the cells [4]. Several members of NIP aquaporins have been shown to function in As(III) uptake, including AtNIP1;1, AtNIP3;1 and AtNIP7;1 in *Arabidopsis*, and OsNIP2;1 in rice [2, 15, 16]. It has been reported that GmNOD26 could be activated by the unknown CPKs in soybean [17], but very little is known about potential signaling mechanisms that regulate these NIP aquaporins. Our study here has demonstrated that CPK31 might target and regulate NIP1;1 for As(III) tolerance, providing the first case for this notion that CPKs regulate As(III) sensitivity

## Supporting information

S1 Table List of primers used in this study. (DOC)
