# Supplementary material for: Calcium-dependent protein kinase CPK31 interacts with arsenic transporter AtNIP1;1 and regulates arsenite uptake in Arabidopsis thaliana
Source: PLoS One. 2017 Mar 15;12(3):e0173681. doi: 10.1371/journal.pone.0173681 (PMC5351991; doi:10.1371/journal.pone.0173681)
Supplement: S1 Table — (DOC) [file pone.0173681.s001.doc]

| **Names** | **Primer sequences** | **Purposes** |
| --- | --- | --- |
| *CPK31*-RT-PCR-F | 5’-ATGGGTTGCTACAGCAGTAAG-3’ | RT-PCR analysis of *CPK31* mRNA |
| *CPK31*-RT-PCR-R | 5’-TCACTTGATCGGAAGAAGCT-3’ |
| pEZS-NIP1;*1*-F | 5'-GGGGTACCAATTTAAACCAAACTTAATAAAATATTACAAGTATGC-3' | Subcellular localization analysis of CPK31 |
| pEZS-*NIP1;1*-R | 5'-TCCCCCGGGAAGTGACTTTTCTCTTTTTAGAAACAAAGAAGAG-3' |
| GUS-F | 5'-GCTCTAGATGCAGCAAATCTATCAGAAGAGGAAAT-3' | Tissue-specific expression analysis of *CPK31* |
| GUS-R | 5’-CGGGATCCTTTCTCAAGAATGGTTCTTTTTGATTGTT-3’ |
| *CPK31*-qPCR-F | 5'-CGCTGGGAGTGCTTACTACATTGC-3' | Q-PCR analysis of *CPK31* expression |
| *CPK31*-qPCR-R | 5'-ACTTCCATGATTCGCTGTCAACGTC-3' |
| *NIP1;1-*qPCR-F | 5’-CCGGTTGTGCATCGGTGGTTG-3’ | Q-PCR analysis of *NIP1;1* expression |
| *NIP1;1-*qPCR-R | 5’- AACGGCCACAAGAGGCGAAG-3’ |
| *ACTIN2*-qPCR-F | 5’-GCCATCCAAGCTGTTCTCTC-3’ | Q-PCR analysis of *ACTIN2* |
| *ACTIN2*-qPCR-R | 5’-GCTCGT AGTCAACAGCAACAA-3’ |
| *NIP1.1-*AD-F | 5’-CGGAATTCATGGCGGATATCTCGGGAAACG-3’ | Y2H analysis of the interaction between NIP1;1and CPK31 |
| *NIP1.1-*AD-R | 5’-CGGGATCCTCAAGTGCTACCGATTCTCACGGT-3’ |
| *CPK31-*AD-F | 5’-CCGGAATTCATGGGTTGCTACAGCAGTAAGAACCT-3’ |
| *CPK31-*AD-R | 5’-CGCGGATCCCTTGATCGGAAGAAGCTCCCTTTG-3’ |
| *NIP1;1-*BD-F | 5’-CCGGAATTCATGGCGGATATCTCGGGAAACG-3’ |
| *NIP1;1-*BD-R | 5’-CGCGGATCCAGTGCTACCGATTCTCACGGTCTTTAG-3’ |
| *CPK31-*BD-F | 5’-GCTCTAGATGCAGCAAATCTATCAGAAGAGGAAAT-3’ |
| CPK31-BD-R | 5’-CGGGATCCTTTCTCAAGAATGGTTCTTTTTGATTGTT-3’ |
| *CPK31-*BIFC3/1-F | 5’-CGCGGATCCATGGGTTGCTACAGCAGTAAGAACCT -3’ | BiFC analysis |
| *CPK31-*BIFC3/1-R | 5’-CGCGGATCCATGGGTTGCTACAGCAGTAAGAACCT -3’ |
| *NIP1;1-BIFC4/2-F* | 5’-TGCTCTAGAATGGCGGATATCTCGGGAAACG -3’ |
| *NIP1;1-BIFC4/2-R* | CGGGGTACCAGTGCTACCGATTCTCACGGTCTTTAG |
